# COVID-19 outbreak, disruption of dental education, and the role of teledentistry

**DOI:** 10.12669/pjms.36.7.3125

**Published:** 2020

**Authors:** Imran Farooq, Saqib Ali, Imran Alam Moheet, Jehan AlHumaid

**Affiliations:** 1 Imran Farooq, Department of Biomedical Dental Sciences, College of Dentistry, Imam Abdulrahman Bin Faisal University, Dammam, Saudi Arabia; 2 Saqib Ali, Department of Biomedical Dental Sciences, College of Dentistry, Imam Abdulrahman Bin Faisal University, Dammam, Saudi Arabia; 3 Imran Alam Moheet, Azra Naheed Dental College, Superior University, Lahore, Pakistan; 4 Jehan AlHumaid, Department of Preventive Dental Sciences, College of Dentistry, Imam Abdulrahman Bin Faisal University, Dammam, Saudi Arabia

**Keywords:** Clinical, COVID-19, Dental education, Didactic, Teledentistry, Patient care

## Abstract

The novel coronavirus disease 2019 (COVID-19) has affected the whole world and has now been declared a Pandemic by the World Health Organization (WHO). Although the mortality rate of this virus is low, it is especially potent against people with underlying systemic conditions. Dentistry is a profession where the doctor, as well as the dental staff, works in close vicinity to the patient’s mouth. Dental education has two core components; didactic and clinical training (including patient care). Dental education has been interrupted in the past due to certain events (Arab Spring and SARS outbreak). Currently, the pandemic of COVID-19 has disrupted dental education globally as most of the dental schools and universities in the world have closed amidst the COVID-19 outbreak. Teledentistry is a subspecialty of telemedicine that helps in the provision of educational activities, advice, and diagnosis about treatment over a distance with the help of technology like video conferencing. The current overview summarizes the potential role of teledentistry in continuing the dental educational process in terms of delivery of didactic components, clinical training, and patient care. It can be concluded that with modern updated devices and tools, teledentistry can be an effective way to prevent disruption of dental education and it can be utilized in continuing the dental educational process in this critical time of the COVID-19 outbreak.

## INTRODUCTION

The novel coronavirus disease 2019 (COVID-19) came into attention after the first case was reported in December 2019 in Wuhan China.^1^ It has since then affected almost all countries in the world and has been declared a Pandemic by the WHO.^2^ This virus belongs to coronavirus family which has similar morphology and structure to severe acute respiratory syndrome (SARS) and the Middle East respiratory syndrome (MERS) viruses.^3^ Although the mortality rate of this virus is low, it is especially potent against people with underlying systemic conditions.^4^ According to a recent study, the median incubation period of this virus is 4.5-5.8 days, and 97.5% people getting this infection will develop symptoms within 11.5 days.^5^ Typical symptoms of COVID-19 include cough, fever, fatigue, diarrhoea, haemoptysis, myaligia, dyspnoea, and pneumonia.^6^ The reported transmission of this disease is from person to person, touching of surfaces contaminated with the virus, or its spread through respiratory droplets.^2^ Currently, work is undergoing to find a cure for this disease but to date, there is no vaccine available for COVID-19.

Dentistry is a profession where the doctor, as well as the dental staff, works in close vicinity to the patient’s mouth.^7^ Most of the treatment procedures in dentistry generate aerosols which could lead to transmission of the virus from patients to the dental staff and vice versa.^8^ Therefore, most of the dental regulatory bodies globally have advised dentists to practice extreme caution while treating patients and provide emergency dental care only in this critical time of the COVID-19 outbreak.^2^ The Bachelor of Dentistry (B.D.S) degree program normally comprises of four years of formal education while in some countries, it can span over a period of six years.^9^ Dental education is usually divided into two parts i.e. pre-clinical and clinical years.^10^ In the pre-clinical years, students are mostly studying basic science subjects didactically and their practical work is restricted to dental laboratories where they practice on the phantom head/extracted teeth.^11^ In the clinical years, students have a bit of didactic part but most of their work is based in the clinical environment, where they work on real patients under the supervision of specialist doctors.^12^

## DISRUPTION OF DENTAL EDUCATION AND TRAINING IN THE PAST

The outbreak of COVID-19 has disrupted dental education globally as most of the dental schools and universities in the world have closed amid COVID-19 pandemic. Unfortunately, this is not the first-time dental education has been disrupted. Few past examples when this happened before are summarized below.

### Disruption of dental education and training during the Arab Spring

In the Arab world, Egypt is one of the countries that has the most number of universities.^13^ In 2011, Egypt faced the Arab Spring due to political unrest in the country.^14^ This resulted in the closure of dental schools and many Malaysian dental students who were studying there were called back by their government.^15^ These students upon their return faced problems like safe return, the difference in the curriculum, high tuition costs, and credit transfer.^15^

### Disruption of dental education and training during the SARS outbreak

In 2003, a new type of pneumonia with high levels of infectivity surfaced globally and was called SARS.^16^ Typical symptoms of SARS included fever, chills, headache, sore throat, dyspnoea, and hypoxia.^17^ Approximately 8,000 active cases of SARS were reported resulting in 800 deaths worldwide.^18^ In March 2003, clinical teaching in the dental hospital of Hong Kong University was immediately ceased.^19^ This decision was based on the emerging reports that 17 medical students from the Faculty of Medicine, Chinese University of Hong Kong had already acquired SARS.^19^ After disruption of almost a month, the didactic teaching resumed on 14^th^ April 2003 whereas the clinical training started 10 days after that in Faculty of Dentistry, Hong Kong University.^20^ It should be noted that, even after the recommencement of educational activities, instructions were given to keep the group teaching to a minimum and to follow strict infection control protocols in the clinics.^20^

## TELEDENTISTRY’S ROLE IN CONTINUING DENTAL EDUCATION AND TRAINING AMID COVID-19 OUTBREAK

From past experiences, it has been learned that dental education is interrupted whenever there is a crisis like war, political unrest, or a disease outbreak. The same situation has ascended with the COVID-19 outbreak where educational institutes (including dental schools) have been closed globally in an effort to restrain the spread of this disease. At this time of crisis, the role of online education becomes very important in dentistry. One synonymous term which was introduced in the past called “teledentistry” has a major role to play in this critical time. In this article, we have used the term teledentistry synonymously with eLearning or online education, whenever dental education is being discussed.

### Origin of teledentistry

Dentistry has seen extensive innovations in technology in the past decade. These innovations include the use of computers, digital diagnostic imaging, digital models, digital planning and designing, and software use for analysis and follow up.^21^ Teledentistry as a subspecialty of telemedicine was introduced in 1994 as part of a military project to improve patient care and dental education by the United States (U.S.) Army.^22^ This project reported that by introducing teledentistry, there was a reduction in patient care costs and it enabled them to extend dental care to distant rural areas.^22^ The term “Teledentistry” could be defined as the provision of advice and diagnosis about treatment over a distance with the help of technology like video conferencing.^23^ Teledentistry can also be used to provide online formal education in the form of web-based self-instructions or interactive video conferencing.^24^ The potential role of teledentistry in continuing dental educational activities (didactic and clinical) amid the COVID-19 outbreak is discussed below in detail.

### Role of teledentistry in delivering didactic component of dental education

**Fig.1:**
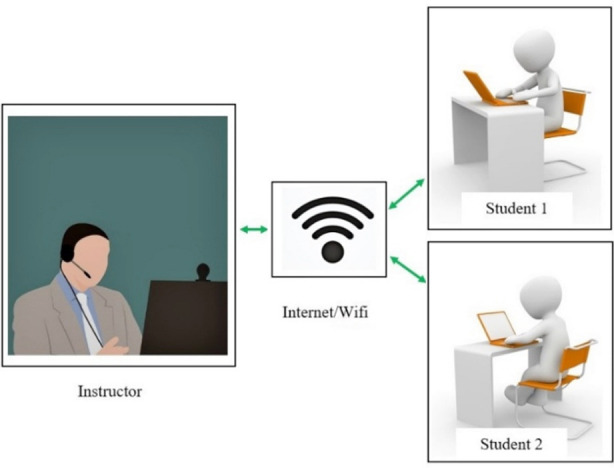
Showing live interactive video lecture technique to cover didactic component of dental education.

**Fig.2:**
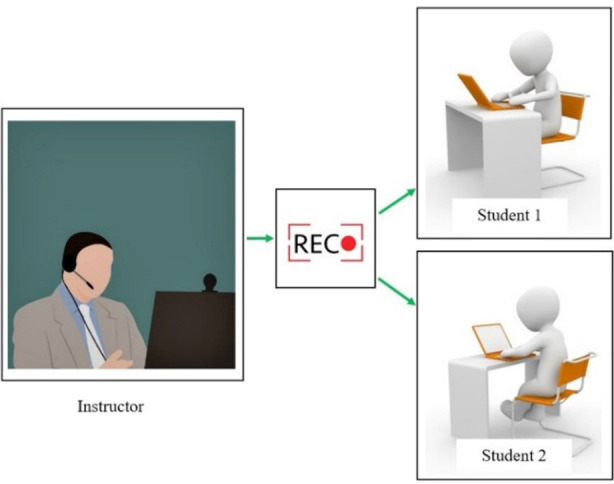
Showing pre-recorded lecture technique to cover didactic component of dental education.

Normally, didactic lectures are attended physically by students and are an essential component of dental education.^25^ It is one of the most popular and commonly utilized for delivering conceptual information to a vast audience at one time.^11^ Unfortunately amid the COVID-19 outbreak, this method of teaching is seriously affected and educational sectors globally are shifting towards online educational tools. At this point, the role of teledentistry becomes indispensable as it can be used to take live interactive video lecture^26^ or a pre-recorded lecture (based on store-and-forward methodology).^21^ The advantages of the former method include live interaction and immediate feedback.^27^ Whereas the latter technique has its own benefits like the user can control the learning pace and can review learning material at their timing of choosing repeatedly.^27^ Through these two methods of teledentistry, lectures can be delivered, instructor and students can have a live interaction, see a demonstration or a prepared stored material, and case discussion can be completed as well.^28^ These two methodologies of teledentistry to deliver the didactic component of dental education are exemplified in Fig.1 and 2.

It is also important to mention that students of the current generation are technology savvy and they prefer online-educational tools over conventional face-to-face lectures.^29^ Farooq and Al-Jandan previously reported that lectures that use modern tools like videos for illustration of phenomenon’s, can enhance students’ learning.^11^ Post-COVID-19 pandemic when all the educational activities are ceased globally, teledentistry could prove to be a vital tool for continuing dental education and training, till the time we return to a normal world where physical distancing is no longer required.

### Role of teledentistry in clinical training component of dental education

Teledentistry can be used to teach few aspects of the clinical training to the students.^21^ Certain clinical subjects in dental curriculum like orthodontics and oral radiology are well suited for teledentistry.^27^, ^30^ In these specialties, it is convenient to collect the patient data and then discuss it with the students via teledentistry tools, even in the physical absence of the patient.^27^ Teledentistry can aid in conducting problem-focused evaluations with students without the need for them to be present.^28^ The use of virtual patients (VPs) in mastering skills like patient interviewing abilities, history taking, and treatment planning has its own importance.^31^ Recently in a randomized controlled clinical trial, Seifert *et al*. (2019), reported that computerized VPs are an effective alternative to lecture led small group discussions in terms of learning effectiveness and learner’s contentment.^32^ In the current scenario where the world is facing COVID-19 pandemic and risk of cross-infection from dental clinics is very high, the use of VPs to teach dental students could be very handy and effective.

### Role of teledentistry in patient care

In the clinical years, dental students are required to treat patients with oral and dental problems under the supervision of subject specialist.^33^ During COVID-19 pandemic, it is need of the hour to triage patients before receiving them for emergency dental care in educational institutions, hospitals, and private clinics through teledentistry tools.^2^ For patient care, teledentistry covers a lot of ground for various dental problems. In prosthodontics, Ignatius E *et al*. (2010) demonstrated the use of video conferencing for prosthetic rehabilitation.^34^ The author reported that with these tools, dental services can be expanded to areas with scarce population as well.^34^ In Oral Medicine, Torres-Pereira *et al*. (2008) has shown that diagnosis of oral lesions can be made via digital images sent through an email.^35^ The author reported that this is an effective tool in diagnosing patients from rural areas where a specialist is not available.^35^ In Oral and Maxillofacial Surgery, it has been previously reported that diagnosis of impacted third molar teeth assisted with telemedicine tools is equivalent to real-time clinical diagnosis.^36^ Teledentistry is also an effective tool in endodontics where diagnosis of peri-apical lesions can be made distantly.^37^ Hence, reducing the cost and number of visits for the patient.^37^ Stephens *et al*. in 2002 reported that orthodontic consultants support teledentistry to make their advice more available to the patients.^38^ The U.S. Department of Defence introduced a system called Total Dental Access (TDA) which helped dentists of US Armed Forces to connect with specialists to discuss patients cases.^39^ This system established that one of the maximum consultations were related to periodontal problems of patients.^39^ In the field of pediatric dentistry, teledentistry has proven its worth as it can be used as a useful tool to assess caries status in young children.^40^

### Limitations of teledentistry

Teledentistry has certain limitations which include a) requirement of a proper internet connection b) the instructor being properly trained in delivering online educational activities and c) limited role to play in clinical training as it mostly involves hand-on practice. Other important considerations while utilizing teledentistry include protection of patient data, involvement of patient in decision making, and referral to appropriate specialist (if needed).

### Limitations of the study

One of the limitations of our study was the inclusion of limited number of articles. However, it should be considered that teledentistry is relatively a new term which is still being evolved. Also, the pandemic of COVID-19 and the role of teledentistry has not been discussed much in the literature. These two points could justify the inclusion of 40 articles in our study. Another limitation was writing a narrative type review, rather than a meta-analysis or systematic review. Again, to defend this, the authors would like to point out towards the scarcity of data available in the current literature highlighting the role of teledentistry in continuing dental education amidst COVID-19 outbreak.

## CONCLUSION

It can be concluded from the current overview that teledentistry can prove to be extremely useful in continuing dental educational activities during the time of COVID-19 outbreak. The current situation has opened the doors for an avenue which was less explored in the past. Teledentistry can help in the deliverance of all essential components of dental education. Teledentistry can also be used in daily practice where it can be beneficial as it would save consultation time for both dentist and patient. In addition, it can be used to triage patients in this critical time of COVID-19 pandemic, before receiving them for emergency dental care in educational institutions, hospitals, and private clinics through teledentistry tools.

### Authors’ Contribution:

**IF:** Conceptualization, Formal Analysis, Investigation, Methodology, Project Administration, Resources, Software, Validation, Writing – Original Draft Preparation, Writing – Review & Editing.

**SA:** Data Curation, Formal Analysis, Investigation, Methodology, Project Administration, Resources, Software, Writing – Original Draft Preparation, Writing – Review & Editing.

**IAM:** Formal Analysis, Investigation, Methodology, Supervision, Validation, Visualization, Writing – Original Draft Preparation, Writing – Review & Editing

**JA:** Formal Analysis, Investigation, Methodology, Supervision, Validation, Visualization, Writing – Original Draft Preparation, Writing – Review & Editing

**IF** takes the responsibility and is accountable for all aspects of the work in ensuring that questions related to the accuracy or integrity of any part of the work are appropriately investigated and resolved.
